# Discriminating Crop, Weeds and Soil Surface with a Terrestrial LIDAR Sensor

**DOI:** 10.3390/s131114662

**Published:** 2013-10-29

**Authors:** Dionisio Andújar, Victor Rueda-Ayala, Hugo Moreno, Joan Ramón Rosell-Polo, Alexandre Escolà, Constantino Valero, Roland Gerhards, César Fernández-Quintanilla, José Dorado, Hans-Werner Griepentrog

**Affiliations:** 1 Department of Weed Science (360b), University of Hohenheim, Stuttgart 70599, Germany; E-Mails: Andujar@uni-hohenheim.de (D.A.); roland.gerhards@uni-hohenheim.de (R.G.); 2 Institute of Agricultural Engineering, Section Instrumentation & Test Engineering (440c), University of Hohenheim, Stuttgart 70599, Germany; E-Mails: hugo.moreno.parrizas@gmail.com (H.M.); hw.griepentrog@uni-hohenheim.de (H.-W.G.); 3 Department of Agricultural and Forest Engineering, Research Group on AgroICT & Precision Agriculture, Universitat de Lleida, Lleida 25198, Spain; E-Mails: jr.rosell@eagrof.udl.cat (J.R.R.-P.); aescola@eagrof.udl.cat (A.E.); 4 Politechnic University of Madrid, E.T.S.I. Agrónomos, Madrid 28040, Spain; E-Mail: constantino.valero@upm.es; 5 Institute of Agricultural Sciences, CSIC, Madrid 28006, Spain; E-Mails: cesar@ica.csic.es (C.F.-Q.); jose.dorado@ica.csic.es (J.D.)

**Keywords:** site-specific weed control, chemical control, weed proximal-sensing

## Abstract

In this study, the evaluation of the accuracy and performance of a light detection and ranging (LIDAR) sensor for vegetation using distance and reflection measurements aiming to detect and discriminate maize plants and weeds from soil surface was done. The study continues a previous work carried out in a maize field in Spain with a LIDAR sensor using exclusively one index, the height profile. The current system uses a combination of the two mentioned indexes. The experiment was carried out in a maize field at growth stage 12–14, at 16 different locations selected to represent the widest possible density of three weeds: *Echinochloa crus-galli* (L.) P.Beauv., *Lamium purpureum* L., *Galium aparine* L.and *Veronica persica* Poir.. A terrestrial LIDAR sensor was mounted on a tripod pointing to the inter-row area, with its horizontal axis and the field of view pointing vertically downwards to the ground, scanning a vertical plane with the potential presence of vegetation. Immediately after the LIDAR data acquisition (distances and reflection measurements), actual heights of plants were estimated using an appropriate methodology. For that purpose, digital images were taken of each sampled area. Data showed a high correlation between LIDAR measured height and actual plant heights (R^2^ = 0.75). Binary logistic regression between weed presence/absence and the sensor readings (LIDAR height and reflection values) was used to validate the accuracy of the sensor. This permitted the discrimination of vegetation from the ground with an accuracy of up to 95%. In addition, a Canonical Discrimination Analysis (CDA) was able to discriminate mostly between soil and vegetation and, to a far lesser extent, between crop and weeds. The studied methodology arises as a good system for weed detection, which in combination with other principles, such as vision-based technologies, could improve the efficiency and accuracy of herbicide spraying.

## 1. Introduction

Current technologies, such as GISand new technical equipment, allow for the possibility of managing the spatial variability of weeds. The concept of site-specific weed management (SSWM) is an attempt to cope with the heterogeneity occurring within fields by treating only weed patches. Herbicides can be applied directly on patches where the weed density is above a previously established threshold [1], or the herbicide application rate can be adjusted to match the weed density. Using SSWM may result in substantial herbicide savings, depending on the weed infestation and its distribution in the field. Gerhards and Christensen Gerhards and Christensen [2], in a four year study with various grass weeds in cereals, showed herbicide reduction higher than 75%. Young *et al.* [3] found an average decrease of 53% in the usage of grass herbicides in wheat. The final implementation of SSWM techniques at a commercial level needs to be seen from the standpoint of the overall prosperity of arable crop production: profitable crop production requires larger investment in new equipment and technologies.

Nowadays, precision agriculture has to find new economic approaches that allow for the managing of sites, specifically, the reality of fields. In this regard, mapping of weeds in agricultural crops can be done by manual sampling, by remote sensing or by sensors located on ground vehicles. Discrete sampling methods have been used in the past for research purposes. However, they are not applicable for practical agriculture [4]. Continuous mapping systems based on visual assessment of weed infestations heavily rely on human perception and have various limitations [5]. Ground-based sensor techniques have shown their potential for weed mapping. Most efforts focus on machine vision techniques to detect and identify plant species [6]. Gerhards and Christensen Gerhards and Christensen [2], using bi-spectral cameras combined with image analysis software, reported a successful differentiation between crops and grass and broad-leaved weeds.

Discrimination between vegetation and soil can be accomplished using optoelectronic sensors [7]. These sensors are not able to distinguish between weeds and crops; thus, they can only be operated in row crops or bare soil. However, this does not represent a major problem if the sensor is operated within the inter-row area. The adoption of these tools highly depends on the cost of weed detection technologies, which is a major deterrent for their commercial introduction. From this point onwards, new approaches have been arising. The use of low-cost sensors could open the door for a reliable adoption of site-specific weed management technologies. Zaman *et al.* [8] developed a real-time sprayer based on ultrasonic sensors. The sprayer was mounted on an ATVvehicle, and it sprayed agrochemicals by activating specific boom sections when weeds were detected.

Andújar *et al.* [9] used a similar system to discriminate broad-leaved weeds from grass weeds according to their height in a mapping approach. These sensors were used for the evaluation of wild blueberries to measure plant height and fruit yield [10]. However, the use of these low-cost sensors has various limitations in relation to their small field of view or footprint. Several sensors are needed for scanning a representative proportion of the field. Indeed, a single sensor with a small footprint cannot scan row and inter-row areas at a time in row crops. In the case of ultrasonic sensors, the main advantages are their robustness and low price, and they have been proven to reliably scan vegetation [11]. Their main drawback is the large angle of divergence of the ultrasonic cone resulting in the largest footprints, which limit the resolution and accuracy of the measurements. Additionally, in this regard, a similar principle with wider scanning possibilities could improve the results.

The use of LIDAR (light detection and ranging) sensing technologies could be a powerful alternative. LIDAR sensors have been widely explored for the geometrical characterization and measurement of biophysical parameters obtained in fruit tree plantations [12]. Together with ultrasonic sensors, they are a reasonably low cost, simple to operate solution [11]. LIDAR technology allows for the scanning of any type of object. Compared to ultrasonic sensors, LIDAR sensors have a wider field of view, a lower divergence of the light beam and gather more data in each reading. They measure the distance between themselves and the objects around them, with a large spatial density of points (resolution) at a very high sampling frequency (thousands of points per second), reconstructing 2D (x,y) and 3D (x, y, z) structures [13,14]. Moreover, LIDAR systems can be accomplished by mounting a source of ultraviolet (UV), visible (VIS) or near-infrared (NIR) light pulses to estimate distances to objects. Additionally, the reflected light pulse intensity together with the type of light could provide some more information on the detected target. The analysis of the reflection value when using NIR light pulses allows for exploiting of an effect that is well known in satellite image analysis: chlorophyll, which is found in living plants, strongly reflects near-IR light [15]. Moreover, the color of an object indicates the reflection value of a point. Bright color shows strong reflectivity [16]. According to this fact, green colors from vegetation could produce high reflection values in the returning laser beams of the LIDAR sensor.

LIDAR technologies have been widely explored when aboard an aircraft or satellites for landscape scanning, but they reach their maximum potential in terrestrial systems [14,17]. Some of the major advantages of ground-based LIDAR sensors are that they are simple to operate, inexpensive and offer high density point clouds. On the other hand, airborne LIDAR scanners are much more expensive and produce data with lower resolution than terrestrial scanners, because of the different point of view and the different laser pulse and geometry used [13]. On-ground applications mainly focus on tree canopy geometric characterization, enabling the implementation of variable rate spraying technologies, among other applications. A good correlation was found between citrus canopy volume and the measurements of a laser scanner [18]. Palacín *et al.* [19] concluded that the foliage surface of fruit trees could be estimated by LIDAR sensing, considering a linear regression between actual and estimated values. On the other hand, Sanz *et al.* [20] showed that both the leaf area and the leaf area density of different hedgerow fruit tree crops, such as apple and pear orchards, and hedgerow vineyards can be successfully inferred from the tree row volume deduced by LIDAR measurements by means of a common logarithmic regression. Andújar *et al.* [21] detected the presence of weeds in maize fields using a LIDAR sensor, allowing for the detection and discrimination of weed groups in the inter-row area. The current study intends to improve those results, using a double index of height and reflection values. In addition, the detection system will work in the row and inter-row area to achieve discrimination between crop and weeds. The possibilities of these devices for object detection and discrimination make them a tool to be explored for weed-crop discrimination.

In this paper, we propose an approach for detecting and discriminating maize plants and weeds from soil surface. We hypothesized that LIDAR sensors could be used for vegetation detection and discrimination, following the results obtained in Andújar *et al.* [21] and looking for an improvement by using a double measurement index. For this purpose, LIDAR heights and reflection measurements were considered. The objectives of this work were: (i) to evaluate the accuracy and performance of a LIDAR sensor for vegetation detection in maize fields; (ii) to assess the possibilities of distance and reflection measurements on vegetation from soil discrimination; and (iii) to evaluate the capabilities of the system for weed-crop-soil discrimination.

## 2. Materials and Methods

### 2.1. Sampling System

A general-purpose laser scanner model LMS-111 (SICK AG, Germany) was used in the data collection system. The measurement principle is based on a 2D divergent laser scanner with a maximum field of view of 270°, with a selectable angular resolution of 0.25° to 0.50°. The emitted laser beams are deflected using a spinning mirror and scan the surroundings in a circular manner. The measurements are triggered at regular angular steps using an angular encoder. The accuracy of the device is ±30 mm in a single-shot measurement, and the standard deviation is 12 mm. The source of light is a pulsed infrared laser of 905 nm wavelength. The distance between the laser scanner and the object of interest is determined according to the time-of-flight principle, by measuring the time delay between an outgoing laser pulse and the returned beam reflected by the impacted object. Moreover, the reflected intensity value was used as the reflectivity. After the laser beam hits the object, it is reflected and travels back to the LIDAR photoreceptor, and an energy loss is incurred. The reflection value returned by the sensor depends on the material of the scanned surface, on the distance at which it is hit and on the angle of incidence. The signal received from a perfectly diffuse reflecting white surface corresponds to a reflection of 100%. Consequently, surfaces that reflect the light as bundled (mirrored surfaces, reflectors) can be assigned a reflection value higher than 100% [22].

The device was positioned to ensure that the 2D point cloud was contained in a vertical plane perpendicular to the crop row, obtaining vertical slices of the crop and weeds. The measurement configuration was set to an angular resolution of 0.25° and a sampling frequency of 25 Hz (40 ms period). The horizontal distance between consecutive measurements in the same scanning plane lied within a range of 2.5–5 mm, depending on the distance between the LIDAR sensor and the scanned object. The minimum object size (Min(*Ø_object_*)) was 20 mm as a function of the beam diameter (*Ø_beam_*), Equation (1), where *d* is the distance in mm between the LIDAR and the object and *D_p_*_−_*_p_*, the distance between the measured points, Equation (2):

(1) $$\emptyset_{\text{beam}}=(d\cdot 0.015\text{rad})+8$$

(2) $$\text{Min}(\emptyset_{\text{object}})=\emptyset_{\text{beam}}+D_{p-p}$$

Readings were obtained by positioning the sensor 60 cm above the ground pointing to the center of the sampled area. Data were acquired from the same stationary location for a 10-s time interval; each sample consisted of approximately 250 scans. The field of view of the LIDAR sensor was set to be from 45° to 135° with respect to the horizontal, which represented a scanned ground width of 1.20 m. Data from the sensor were transferred to the computer via an Ethernet communication port. SOPASsoftware (SICK Open Portal for Applications and Systems Engineering Tool) was used to control the sensor and store data in the computer. Raw data collected by the LIDAR sensor were configured in dual mode output: distance and reflection. The sensor provided the radial distance corresponding to each angular direction of laser beams (polar coordinates) and the respective reflection value, *i.e.*, each point was characterized by the distance and an angle referred to as 0°, corresponding to the center of the LIDAR. By the distance extraction, a height profile was obtained containing all the points where the laser beam impacted. Each profile was composed of the points of intersection between the laser beam and the vegetation or soil present in the row and inter-row sampled area. From the sensor return, a second profile contained the reflection values at each point.

### 2.2. Site Location and Data Processing

Field measurements were conducted on June 5th and 6th, 2012, on maize. The field trial was located in SW Germany, at the experimental station, Ihinger Hof (48°7′ N, 8°9′ E; altitude 450 m a.s.l.), in Renningen, University of Hohenheim. This place has around 715 mm mean annual precipitation and a mean temperature of 9.3 °C; the soil is of a loam type (loess weathered soil). Precipitation is approximately equally distributed over the year with a peak in summer and a minimum in winter. Maize was planted on April 12th at a 0.75 m inter-row distance and a density of 90,000 plants • ha^−1^. The measurements were carried out on a maize growth stage BBCH12-14. The maize field was infested with *Echinochloa crus-galli* (L.) P.Beauv, *Lamium purpureum* L.and *Galium aparine* L., but also included weed-free areas. The field was sprayed with a pre-emergence treatment of S-metolachlor and mesotrione.

**Figure 1.**
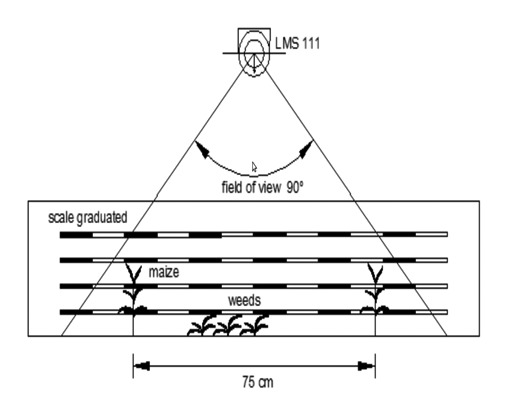
Schematic design of the sampling methodology at scanning position.

Residual weed patches were abundant; thus, 16 samples were chosen, looking for different weed density ranges in order to assess representative measurements. Densities ranged from 0 (bare soil) to >100 plants · m^−2^. The number of samples were previously calculated according to CSIC [23]. In some cases, it was necessary to hand-weed the plots, in order to obtain a representative number of samples for the statistical analysis. Immediately after the LIDAR sensor data collection, the actual height of plants was assessed. For that purpose, RGBimages were taken of each sampled area following the procedure proposed by Andújar *et al.* [21], frontally pointing a camera held at a height of 20 cm. The camera was a Canon digital EOS 5D (Canon Inc, Tokyo, Japan) with a 6.1 megapixel DXFormat CCDimage sensor equipped with an 18–70 mm lens. A frame with a graduated scale in centimeters was located on the back of the sample and placed with its vertical plane perpendicular to the LIDAR axis (Figure 1). The collected images were processed in advance in the lab.

Raw data were processed off line in an application developed in Matlab® 2010 (The Mathworks Inc., Natick, MA, USA). The software was able to manage and plot the stored data, creating a plane with the associated values of LIDAR heights and reflection. The scan data included the scan angle, range and reflection values. Thus, a plane of heights was created with the associated values of reflection. The data, transformed from polar to Cartesian coordinates, were exported to AUTOCAD 2010 (Autodesk Inc., Mill Valley, CA, USA). As a result, two 2D point clouds per sample (geometrical profile and reflection profile) were plotted. Afterwards, the frontal images were projected into the same file. This procedure allowed for manually drawing the actual profiles over the RGB images, as they were scaled. This methodology was repeated for each sample. Consequently, three 2D profiles were visible for each sample: actual height, LIDAR height and reflection values. Each profile was graduated in centimeters and cropped, obtaining a database with values for these three parameters. Then, a matrix for each file was created, containing all values related to actual height, LIDAR height and reflection values.

### 2.3. Statistical Analysis

Regression analyses were applied to identify the capabilities of the system to discriminate between vegetation and soil. For that purpose, two cases were considered: (i) the geometrical reliability of the LIDAR, considering a linear relationship between the actual plant heights and the height values measured with the LIDAR sensor; and (ii) the ability of the system to discriminate the presence/absence of vegetation. Due to the fact that presence/absence is a dichotomous variable, a logistic binary regression was used. Logistic regression is able to predict the presence or absence of a characteristic based on values of a set of predictor variables. The coefficients can be used to estimate odds ratios for each of the independent variables in the model. Logistic regression does not make many of the key assumptions of linear regression that are based on ordinary least squares algorithms regarding linearity, normality, homoscedasticity and measurement level. It does not need a linear relationship between the dependent and independent variables, which do not need to be multivariate normal. In addition, the residuals do not need to be multivariate normally distributed, and homoscedasticity is not needed. However, some other assumptions, like a binary-dependent variable and an ordinal independent variable, are needed. The errors needs to be independent [24]. This analysis employs binomial probability theories, in which there are only two values to predict: that probability (p) is 1 rather than 0, *i.e.*, the event (vegetation/soil) belongs to one group rather than to the other. A backward stepwise logistic regression was carried out.

The parameters for predicting the outcome of a dependent variable, presence/absence (vegetation or soil), was based on two predictor variables: LIDAR height and reflection values. After testing the normal distribution of the two independent variables, LIDAR heights and reflection values, analyses were performed to assess the capability of independent variables to discriminate significantly between vegetation and soil. Indeed, the possibility of weed type-crop-soil discrimination was tested with Canonical Discriminant Analysis (CDA). Discriminant analysis builds a predictive model for group membership. The model is composed of a discriminant function or a set of discriminant functions based on linear combinations of the predictor variables that provide the best discrimination between the groups. The functions are generated from a sample of cases for which group membership is known, and the functions can then be applied to new cases that have measurements for the predictor variables [24]. This methodology is used for pattern recognition to find a linear combination of features, which can separate two or more groups based on the variables characterizing the dependent variable. This method expresses the dependent variable as a linear discriminant combination of the other features [25]. Due to overlapping in the multivariate space, Monte Carlo permutation was used to calculate the probability of occurrence of a point into a certain class. Monte Carlo permutation is a Bayesian method that uses pseudo-random (computer simulated) values to estimate the probability of occurrence, using a large set of random samples of state variable values [26]. In this study, two cases were analyzed: (I) the possibilities of the method to separate vegetation areas from bare soil; and (II) the possibilities of the discrimination of four types of situations: (a) monocots; (b) dicots; (c) crop; and (d) soil. Analyses were carried out with the software SPSS v20 (IBM SPSS Statistics).

## 3. Results

**Figure 2.**
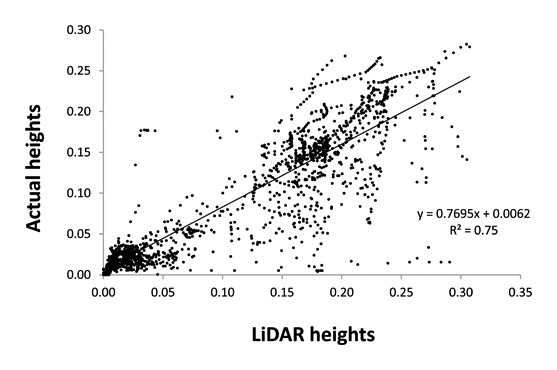
Linear regression between actual plant height (m) and LIDAR measured height.

**Figure 3.**
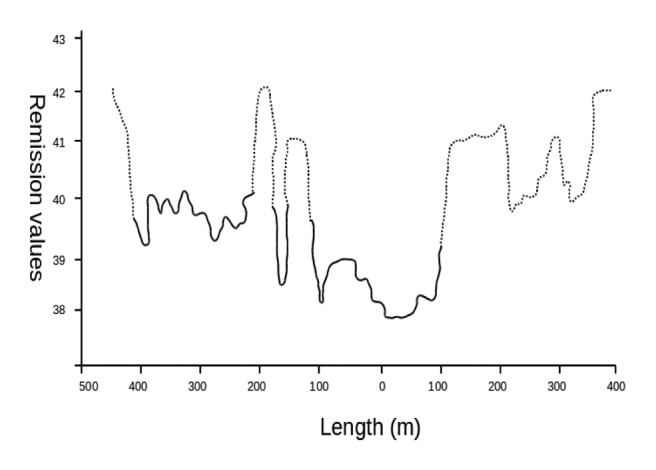
Reflection measurement for soil, weeds and maize. A continuous line (—) represents soil presence. A dotted line (- - -) represents vegetation presence.

A linear relationship between LIDAR measured heights and actual plant heights was found, proving the accuracy of the method for plant height determination. The results showed a strong correlation between actual heights and LIDAR heights, with an R^2^ = 0.75 (Figure 2). However, actual height, as well as weed densities were not well correlated with LIDAR reflection values (data not shown). Although the reflection values did not show a high relationship with heights (LIDAR and actual heights), the values could reproduce the vegetation profile (Figure 3), and its use could improve the accuracy. Furthermore, this study proves that bright colors (green color from vegetation) belong to high reflection values accentuated by the chlorophyll activity. Thus, the reflection value represents object information close to the intensity of color.

Figure 3 shows an example of the reflection plane identifying the vegetation areas (maize and weeds) and bare soil. It is clear that higher reflection values correspond to vegetation presence. The logistic regression showed a high accuracy in the LIDAR measurements for predicting the presence or absence of vegetation. The reflection values used to reinforce the distance measurements improved the vegetation prediction. From a total of 1,558 sampling units, the predicted values showed an accuracy of 95.3% for vegetation and 82.2% for soil (or non-vegetation), with an overall accuracy of 92.7% (Table 1). Thus, the results from the logistic binary regression showed that the procedure was highly accurate for plant detection. The ANOVA test showed that there were significant differences between groups.

**Figure 4.**
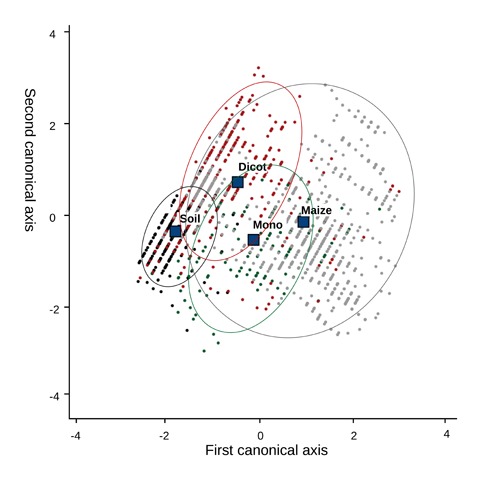
Canonical discriminant function plot of LIDAR measurements representing four groups: monocots, dicots, crop and soil.

The CDA could separate between vegetation areas from bare soil with a lower success than the logistic regression. However, the analysis proved that the discrimination between four types of situations, (a) monocots; (b) dicots; (c) crop and (d) soil, was possible, with an overall success of 72.2%. The cases of soil (d) were correctly classified with 92.4% of accuracy. Dicots (b) were properly classified in 64.5% of the analyzed cases, with all misclassified cases distributed between the other groups. The presence of monocots (a) was poorly classified, mainly because it was identified as crop. In addition, the predictions for crop (c) presence showed good results, with 74.3% of cases integrated in the right group (Table 2). The canonical discriminant function plot (Figure 4) identified differences between groups. The centroid of soil (d) was clearly separated from the rest of the centroids. In addition, the centroid of dicots (b) was clear from the rest of the groups, with some overlapping with crop (c) and monocot (a) groups. The centroid of these two groups, crop (c) and monocots (a), presented some discrimination. However, some overlapping was present between them, containing some of the misclassified samples.

According to the observed frequency in the measured data, most of the points corresponded to maize plants (50.64%). Soil represented 20.15% of the points, dicots 19.7% and monocots 9.5%. Table 3 shows the results based on Monte Carlo simulation of 10,000 iterations. The obtained values were quite similar to the observed ones. It was concluded that these samples were sufficient to make a robust inference, and as such, it was not necessary to run the chain for longer.

## 4. Discussion

According to the results of this system for weed and crop detection, we can state that 905-nm wavelength LIDAR is a reliable principle for plant detection. The readings from the sensor and the actual height of the vegetation were quite similar, these values being highly correlated. A similar conclusion was reached in a study using ultrasonic sensors to discriminate broad-leaved weeds from grass weeds [9]. A significant correlation between the actual heights of different weed species and ultrasonic measurements was found. Although ultrasonic devices can measure plant heights with high accuracy, they are not able to create a plane in a single reading, because their output is a unique value corresponding to the field of view (FOV). A LIDAR sensor creates a continuous plane containing multiple values for weed and crop height. In the case of row crops, such as maize, crop rows would be easily identified by using the crop position in the plane, and more efforts could be focused on weed discrimination in the inter-row area. In addition, the resolution of LIDAR sensors is higher than the one of ultrasonic sensors, because of its smaller laser beam footprints, high angular resolution and wider field of view.

The logistic regression showed promising results, with more than 92% of the cases properly classified. This shows the high capabilities of the system for discrimination between crop and weeds with a double index. In the previous study by Andújar *et al.* [21], the discrimination system was focused on the inter-row area, and crop plants had to be manually removed from the sample. The use of the double index allowed us to discriminate crop and weeds. The errors could be due to the structural complexities and variations of maize and weeds and the size of the laser beam. The bigger the size of the laser beam footprint, the lower the resolution. Hence, a higher accuracy would decrease the field of view, which would limit the applicability of the method. In the current method, the use of reflection improved the results. Compared to machine vision techniques, the reflection information could be used more reliably than color intensity, because the value is hardly affected by outdoor weather or brightness conditions [27]. Unfortunately, the reflection values returned by the scanner depend on the material of the scanned surface, on the distance at which it is hit and on the angle of incidence [28], with a non-linear relationship [29].

The brightest colors (vegetation) cause higher reflection values. However, some soil components could have high reflection values, as well, which make the process of vegetation extraction much more challenging than the ideal case. One of the reasons can be the breeze while measuring, as it affects LIDAR measurements, because of the change that the crop and weed leaves pose. This implies that the laser beam, instead of aiming at the leaves, is hitting the soil surface. As a result, the measurement is wrong. The height of 60 cm over the soil was chosen in order to minimize the error in the measurement (minimum object size: 20 mm), e.g., the laser beam spot can hit half leaves and the other half, soil. Consequently, the signal received by the LIDAR is the average reflection value of the soil and the plant. Nevertheless, this situation can be overcome, since some LIDAR sensors are able to provide different echoes for partially occluded laser beams, so that the data collection system would know if the beam partially hit a leaf and continued to another leaf or to the ground. In this way, the reflection value could be corrected or removed from the analysis. In addition, when the laser beam is perpendicularly incident on a surface, the energy is optimal. In some cases, the reflection values from vegetation and soil were really close. Therefore, reflection data together with LIDAR height measurements were used to robustly distinguish between soil and vegetation.

The capability of weed discrimination was proven by the CDA analysis, with a success of 72.2%. The proportion of properly classified cases was 92.4% for bare soil, with misclassified cases belonging mainly to dicots, which were shorter than the crop and grasses. This result shows the influence of the reflection index to improve the discrimination. In Andújar *et al.* [21], the CDA could not discriminate correctly among groups. In addition, maize crop was well classified in 74.3% of the studied cases, with all misclassified data distributed within the other categories. Although, the crop discrimination reached a good prediction value, the crop identification could be improved by a positioning algorithm, because crop rows remain at a constant distance between them. This could be used for a double purpose: in the first instance, for weed identification and, secondly, for robot guidance in the case of autonomous vehicles, which could follow the crop row. A similar device was explored in three crops as an auxiliary element used to provide feedback on the steering angle, with an average error of 2.5 cm [30], which shows the accuracy of these systems.

The case of weed discrimination showed poor results for monocots, due to its similarity to maize plants. However, this effect would be easy to avoid if the sensor readings are exclusively analyzed in the inter-row area. In addition, the possibility of multiple sensors data fusion should be explored. A double output could lead to an on-line system for weed-crop-soil identification. The use of low-cost cameras could solve the row identification process [31], and LIDAR measurements could be used exclusively for weed detection purposes. This new approach of high accuracy, stationary measurements for weed discrimination in a 2D stage could be implemented in 3D following the principles established for tree crops characterization, *i.e.*, multiple scans from different points [11,14]. The sensor could be mounted on a ground vehicle scanning in a continuous way in combination with an inertial measurement unit, to compensate for the vehicle movements. Although the sensor used is a 2D scanner, this displacement in the forward direction at a constant speed would allow us to build up a 3D profile of the field.

## 5. Conclusions

Ongoing research is looking for new and improved tools for weed detection. The solutions require high accuracy and fast response at a low cost. However, nowadays, these characteristics are not present in any commercial system. NIR LIDAR technology has been probed for its capability for weed-crop-soil discrimination with promising results by itself or in combination with other sensors. In addition, the information provided by the sensor could be used for a double purpose: detection and guidance, if the LIDAR sensor is mounted on an autonomous vehicle.

## Figures and Tables

**Table 1.** Binary logistic regression values showing the percentages of classification using light detection and ranging (LIDAR) height and reflection values.

**Observed**	**Predicted**	

	**Soil**	**Vegetation**
Soil	82.2	17.8
Vegetation	4.7	95.3

**Table 2.** Percentages of the original classes correctly classified by the Canonical Discrimination Analysis (CDA) in four predefined groups based on LIDAR measurements.

	**Predicted Group**			

	**Soil**	**Monocots**	**Dicots**	**Crop**
Soil	92.4	1.6	6.1	0
Monocots	19.6	34.5	14.9	31.1
Dicots	28.2	6.2	64.5	11.1
Crop	4.8	8	12.9	14.4

**Table 3.** Observed and simulated Monte Carlo occurrence of monocots, dicots, crop and soil, including 95%-confidence intervals (95%-CI).

**Frequency**	**Observed**		**MC Simulation**	
	
	**Relative**	**Absolute**	**Estimate**	**95%-CI**
Soil	0.2015	0.2015	0.1984	0.00–0.19
Monocot	0.0950	0.2965	0.0915	0.20–0.28
Dicot	0.1970	0.4936	0.1976	0.29–0.48
Maize	0.5064	1.0000	0.5125	0.49–1.00
